# Supplementary material for: Human-induced westerly jet shifts coordinate terrestrial productivity at the hemispheric scale
Source: Nat Commun. 2026 Jun 4;17:4960. doi: 10.1038/s41467-026-74039-3 (PMC13236986; doi:10.1038/s41467-026-74039-3)
Supplement: Supplementary file 1 — Supplementary Information [file 41467_2026_74039_MOESM1_ESM.pdf]

Supplementary Material for

Human-Induced Westerly Jet Shifts Coordinate Terrestrial Productivity at the Hemispheric Scale

Xiaoye Yang, Aiguo Dai, Gabriele Messori, Bin He, Zhibo Li, Ziqian Zhong, Xing Yuan, Chang-Hoi Ho, Dim Coumou, Botao Zhou, Deliang Chen*

Corresponding author: Deliang Chen (deliangchen@tsinghua.edu.cn)

This file includes:

Supplementary Notes

Figures S1 to S16

Supplementary Notes

Causal pathways of westerly curvature regulating gross primary productivity in the mid-to-high latitudes of the Northern Hemisphere

Within our framework, we emphasize treating large-scale westerly circulation changes as an organizing dynamical mode that can restructure regional climate conditions and thereby regulate spatial patterns of GPP, while also attempting, to some extent, to explicitly identify the direct dynamical effects of westerly jet variability from indirect pathways mediated through hydroclimate or land–atmosphere interactions.

Here we introduc Liang-Kleeman Information Flow Theory[1,2]. Compared with the traditional Granger causality approach, the information flow approach provides a more physically interpretable measure of causality, as it is derived from dynamical system theory and quantifies the directional transfer of information between variables. Unlike Granger causality, which is primarily based on predictive capability within linear statistical models, the information flow approach is better suited for characterizing complex nonlinear interactions and stochastic dynamics in climate systems[3,4]. Using this theory, the causal relationship can be determined only by the time series of the two parameters. According to the theory, under the assumption of linear mode, the maximum likelihood estimation form of the information flow from X_2 to X_1 (X_1 and X_2 are two time series) is:

$$T_{2\rightarrow1} = \frac{C_{11}C_{12}C_{2,d1} - C_{12}^2 C_{1,d1}}{C_{11}^2 C_{22} - C_{11}C_{12}^2}$$

where C_{ij} is the covariance of X_i and X_j and $C_{i,dj}$ is the covariance of X_i and $\{(X_{j,n+1} - X_{j,n})/\delta t\}$, with δt represents the time interval. According to the theory, causation implies correlation. However, the converse is that correlation does not imply causation. When $|T_{2\rightarrow1}| > 0$, X_2 is a cause of X_1; and when $|T_{2\rightarrow1}| = 0$, X_2 is not the cause of X_1. $|T_{2\rightarrow1}|$ represents the information flow strength from X_2 to X_1, with larger values indicating stronger causal associations. To assess causal strength in a multivariate system, we used standardized information flow. LK information flow can be computed using the Python package LK-Info-Flow (https://pypi.org/project/LK-Info-Flow/).

We applied the information flow approach to quantify the mutual information flow strength among westerly curvature, circulation factors (200 hPa geopotential height, zonal and meridional winds), local climate variables (surface maximum temperature and precipitation), and GPP within five key sectors:

Eastern Europe, Central Asia, East Asia, Western North America, and Central North America. Based on the information flow matrices (Extended Figure 7a), we constructed a critical causal network to further identify the pathways through which westerly curvature influences terrestrial GPP (Extended Figure 7b). Specifically, we extracted causal connections with standardized information flow strength greater than 0.1, corresponding to approximately the top 60% of all possible connections, to highlight the most significant causal pathways.

In this causal network (Extended Figure 7), the role of westerly curvature can be interpreted as an upstream dynamical regulator within a coupled "circulation dynamics–surface (hydro-) climate factors–GPP response" cascade. Its influence on GPP is not realized through a single pathway, but instead emerges from multiple interacting and nonlinearly coupled transmission routes. Structurally, westerly curvature acts as a diagnostic metric of Rossby wave morphology. By modulating jet stream meandering and ridge–trough configurations, it is significantly associated with the 200-hPa geopotential height ($T_{\text{westerly curvature}\rightarrow z_{200}}$=0.24) and meridional wind fields ($T_{\text{westerly curvature}\rightarrow V_{200}}$=0.19). This indicates that geometric changes in the mid- to high-latitude circulation induce a systematic dynamical reorganization, leading to enhanced waviness of the large-scale flow and strengthened meridional transport. Building on this dynamical adjustment, anomalies in geopotential height and meridional wind jointly regulate surface hydroclimatic conditions, thereby reshaping regional energy balance and moisture transport pathways. At the climate response level, circulation anomalies exert a pronounced influence on two key ecological limiting factors: maximum surface air temperature ($T_{z_{200}\rightarrow T_{max}}$=0.20) and precipitation ($T_{z_{200}\rightarrow \text{Precipitation}}$=0.14; $T_{V_{200}\rightarrow \text{Precipitation}}$=0.17). Importantly, temperature and precipitation do not respond independently; instead, they exhibit coupled responses under the same large-scale circulation background ($T_{T_{max}\rightarrow \text{Precipitation}}$=0.36). At the GPP response level, GPP is jointly constrained by both precipitation and temperature, although the precipitation limitation is more dominant ($T_{\text{Precipitation}\rightarrow \text{GPP}}$=0.33). Meanwhile, temperature plays a secondary regulatory role by influencing evapotranspiration demand and other physiological requirements for vegetation growth ($T_{T_{max}\rightarrow \text{GPP}}$=0.23).

Therefore, the whole structure of the causal network reveals a clear yet highly coupled propagation chain: west wind curvature, as an upstream dynamical modulator, reshapes the upper-level circulation, subsequently affects key (hydro-) climatic variables such as temperature and precipitation, and then

regulates variations in GPP ($T_{\text{westerly curvature}\rightarrow GPP}$=0.26). More importantly, the inferred network reflects not merely a linear cascade, but a multilevel system characterized by feedback coupling. West wind curvature not only influences downstream variables through geopotential height and meridional wind, but these climatic variables also interact with each other. As a result, changes in thermal and hydrological conditions are not simply additive; rather, they are systematically amplified or suppressed through dynamic–thermodynamic coupling, highlighting the presence of complex nonlinear interactions within the climate system.

Supplementary Figures

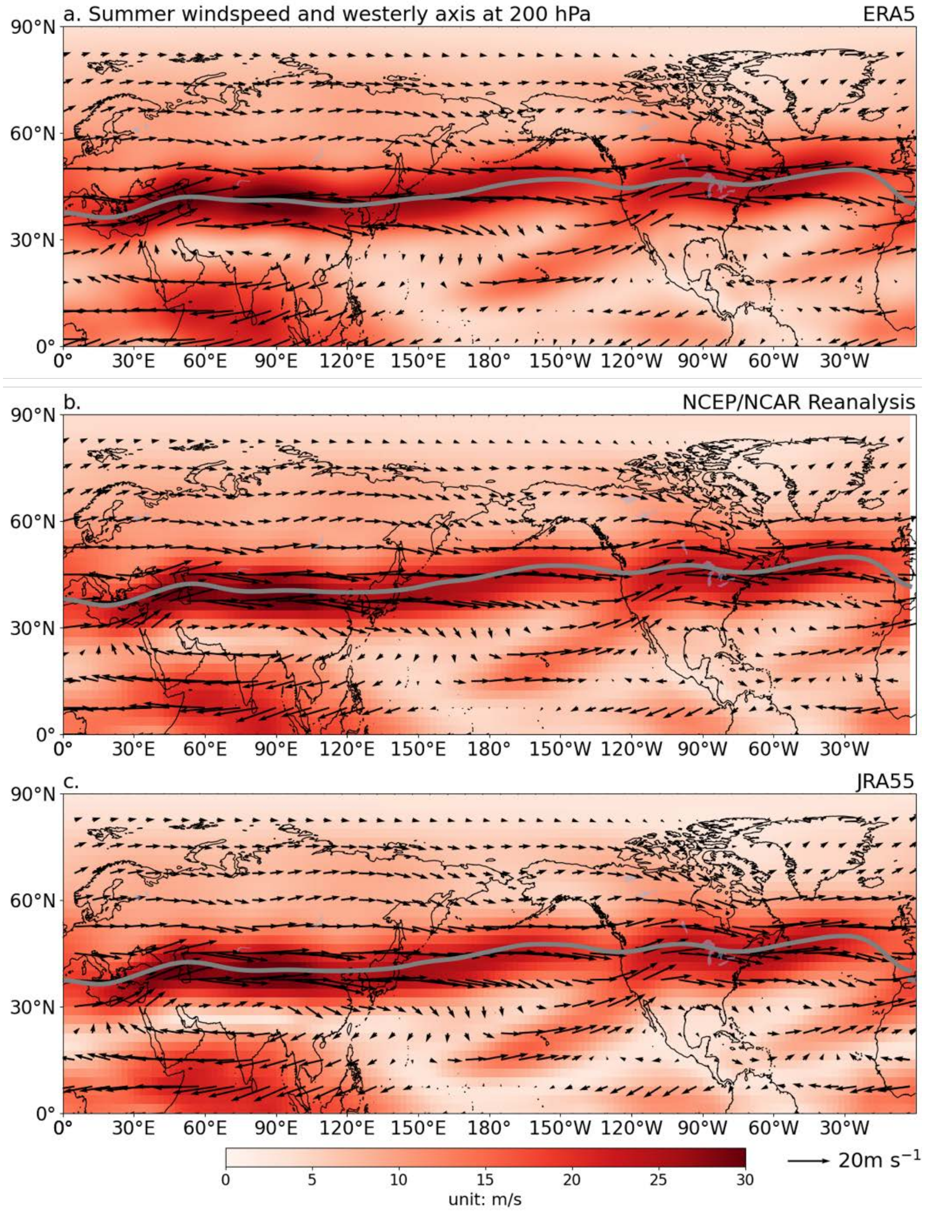

Figure S1. Summer mean wind speed (shading; m s^{-1}), horizontal wind (vectors; m s^{-1}), and westerly jet axis (gray line) at 200 hPa over the Northern Hemisphere, averaged for 1979–2023, derived from (**a**) ERA5, (**b**) NCEP–NCAR Reanalysis, and (**c**) JRA55. Spatial correlation coefficients between datasets are significant at the 99% confidence level.

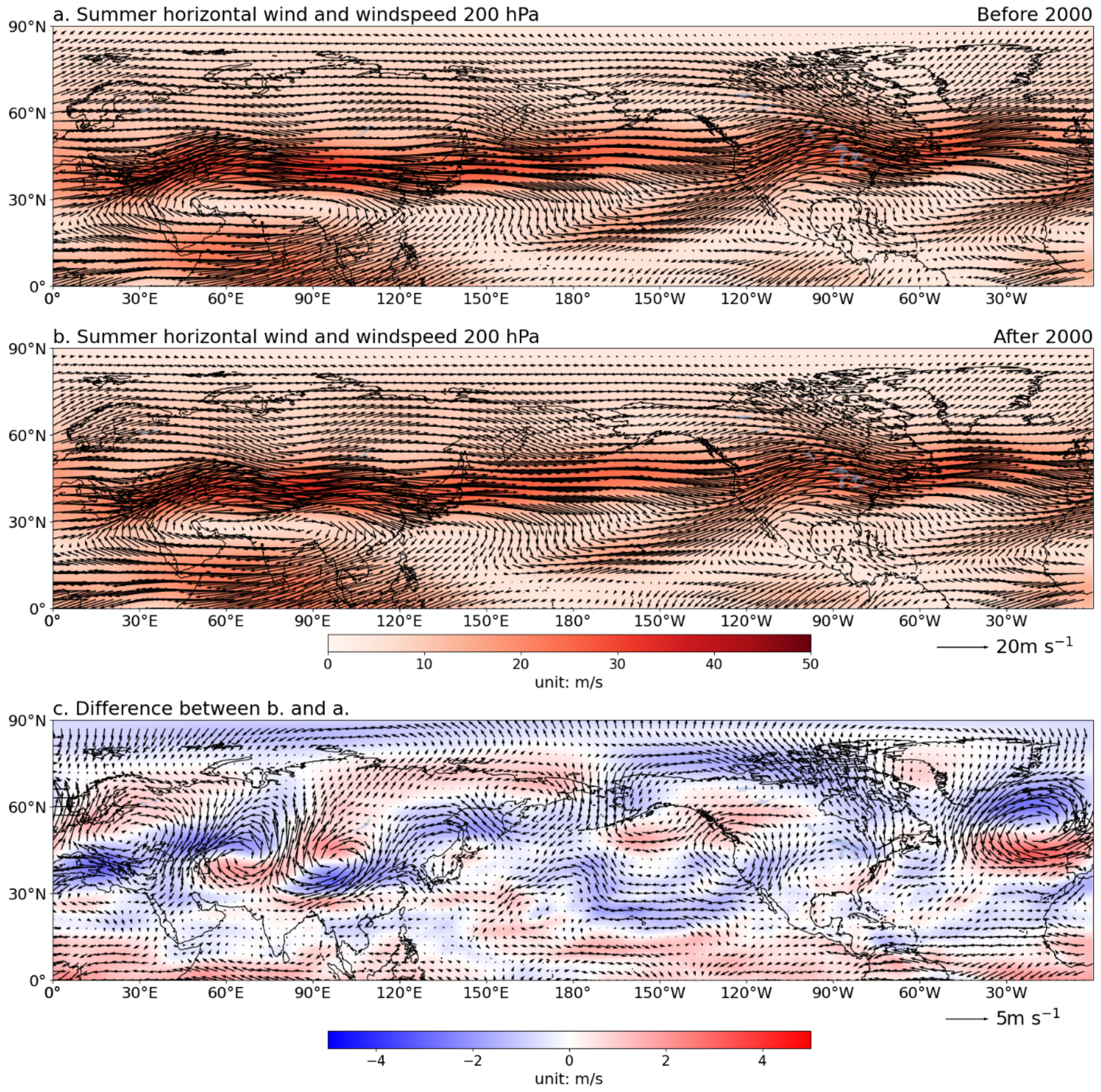

Figure S2. Summer mean wind speed (shading; m s^{-1}) and horizontal wind (vectors; m s^{-1}) at 200 hPa over the Northern Hemisphere, averaged for (**a**) 1979–1999 and (**b**) 2000–2023, and (**c**) their difference (**b** minus **a**).

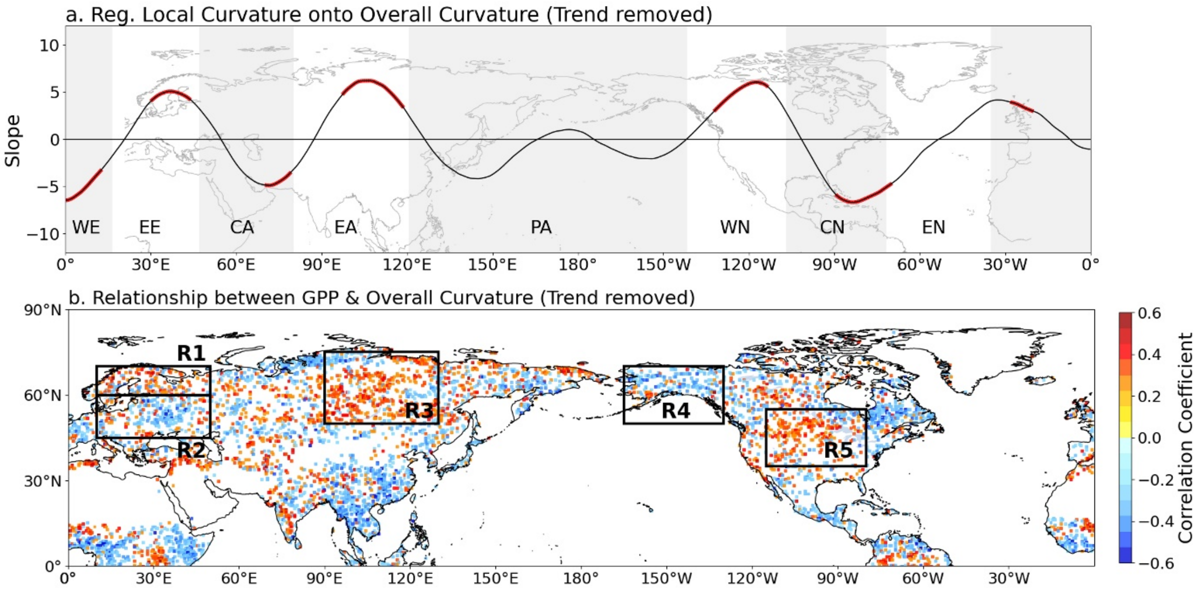

Figure S3. Westerly curvature and its relationship with GPP. (a) Linear regression coefficients of local westerly curvature onto the overall Northern Hemisphere curvature along longitude, with the linear trend removed. Red-shaded areas indicate statistical significance at the 95% confidence level. (**b**) Spatial distribution of correlation coefficients between local summer GPP and Northern Hemisphere overall curvature (1982–2018) after removing the linear trend. Only correlations significant at the 95% confidence level are shown.

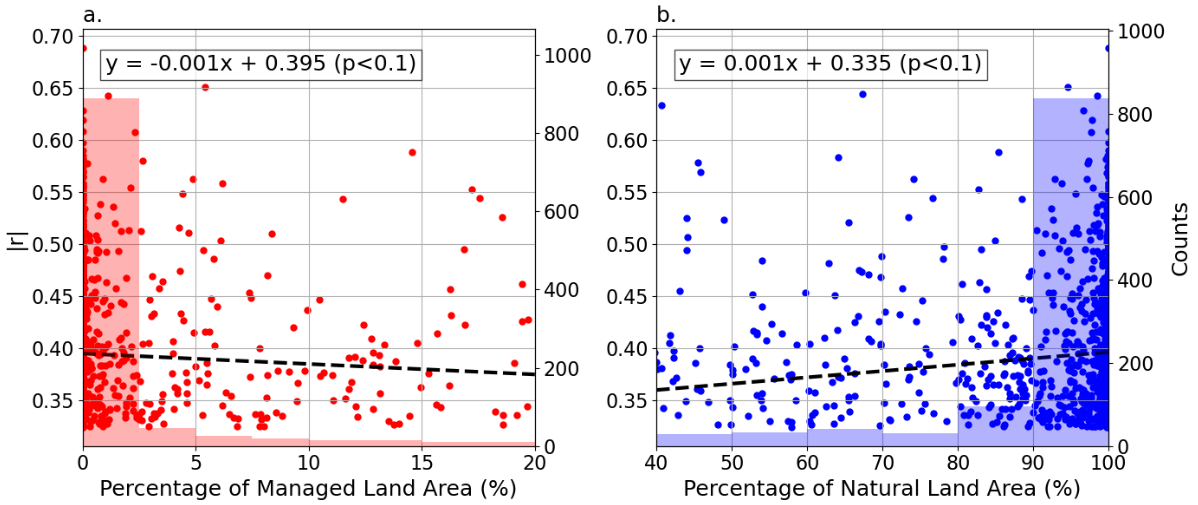

Figure S4. Influence of land-use type on the relationship between westerly jet curvature and GPP. Joint distributions of **(a)** managed land fraction (%) and **(b)** natural land fraction (%) with the absolute correlation between westerly jet curvature and GPP (|r|). Overlaid histograms show the number of grid cells in each |r| bin, and black dashed lines indicate linear regression fits.

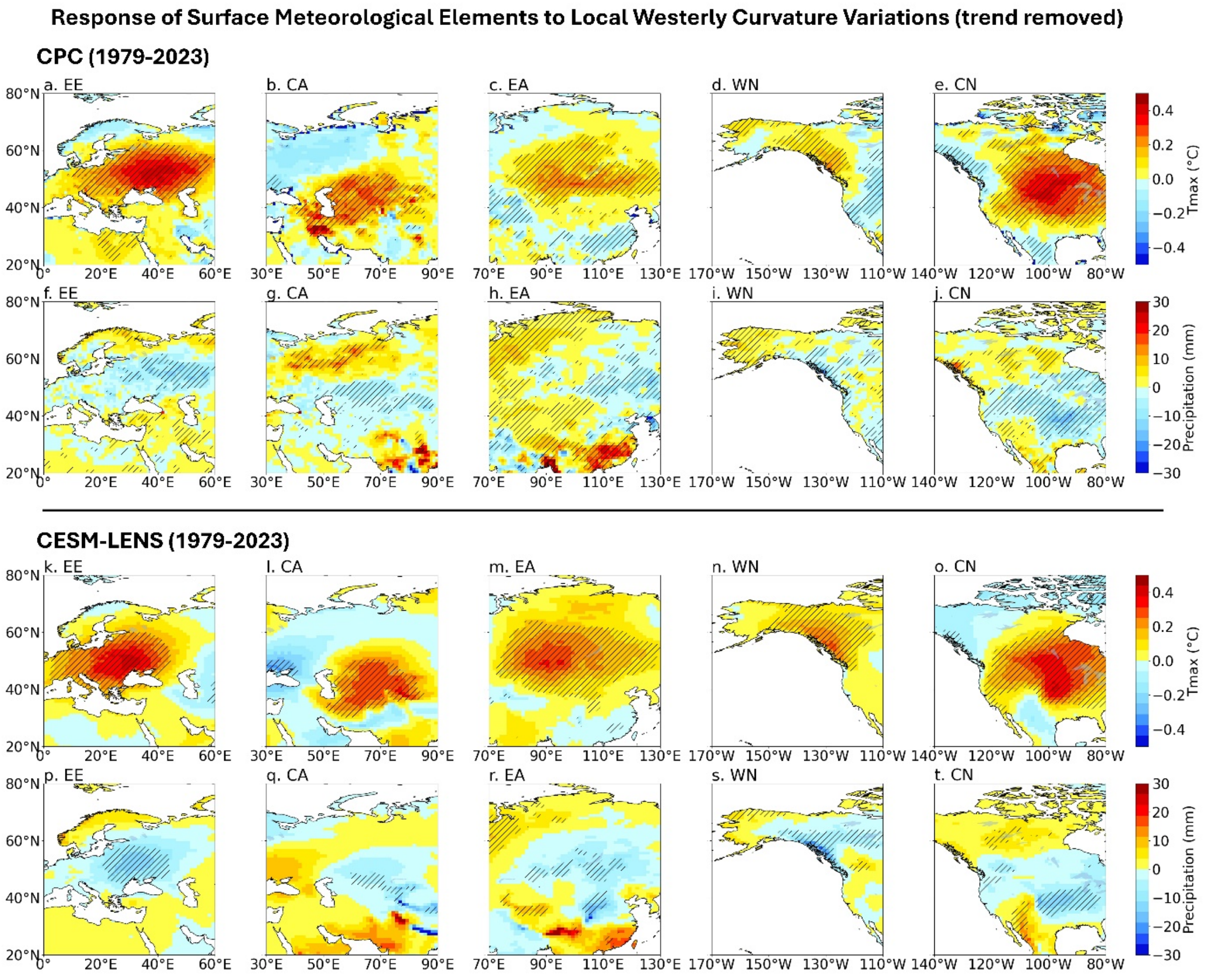

Figure S5. Response of surface meteorological elements to local westerly curvature variations. Linear regression of surface meteorological variables onto local westerly jet curvature (1979–2023) in five regions: Eastern Europe (EE), Central Asia (CA), Eastern Asia (EA), Western North America (WN), and Central North America (CN). (**a–e**) Regressions of maximum surface air temperature (Tmax, °C) based on CPC observations; (**f–j**) regressions of precipitation (mm day^{-1}) from CPC. (**k–o**) Tmax and (**p–t**) precipitation regressions derived from CESM-LENS simulations.

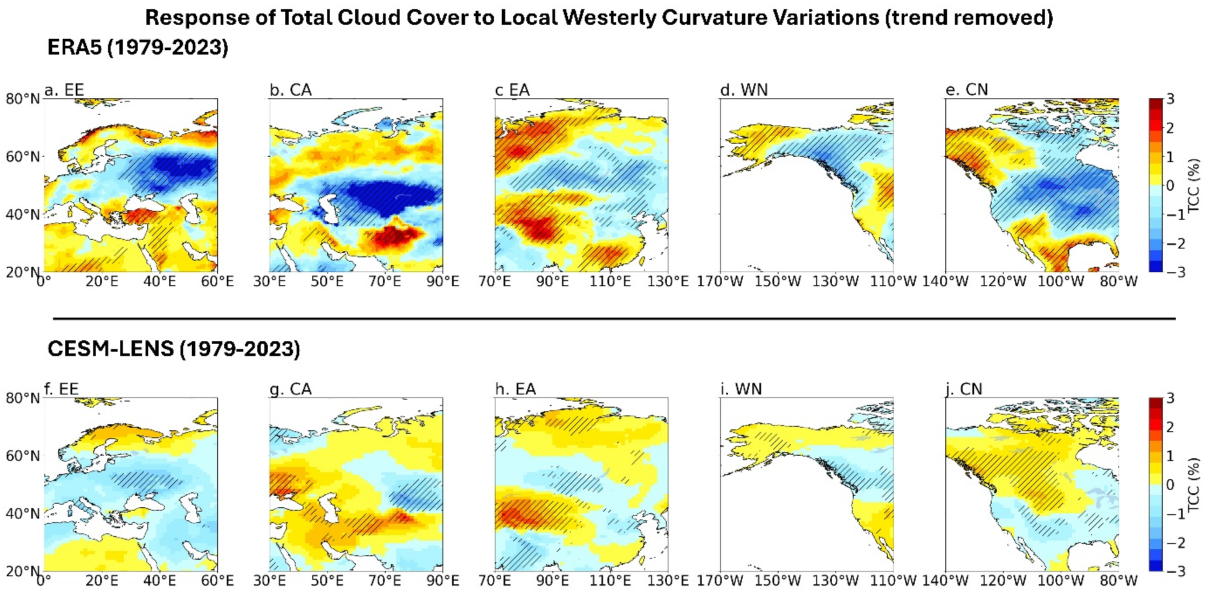

Figure S6. Response of total cloud cover to local westerly curvature variations. Linear regression of total cloud cover (TCC, %) onto local westerly jet curvature (1979–2023) in five regions: Eastern Europe (EE), Central Asia (CA), Eastern Asia (EA), Western North America (WN), and Central North America (CN). (**a–e**) Regressions based on ERA5 observations; (**f–j**) regressions derived from CESM-LENS simulations.

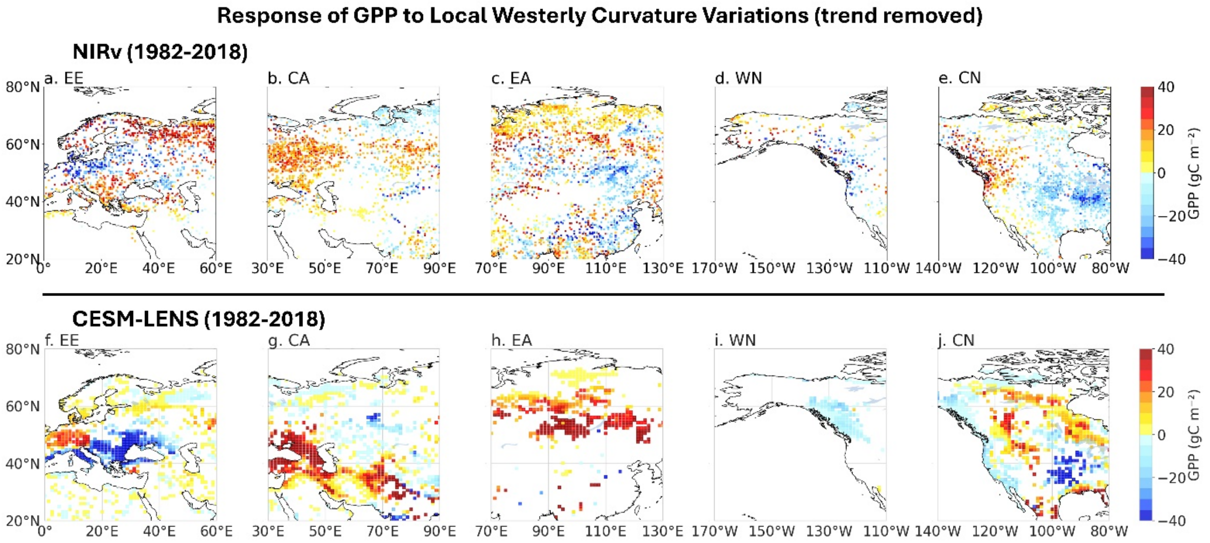

Figure S7. Response of GPP to local westerly curvature variations. Linear regression of Gross Primary Productivity (GPP, g C m^{-2} yr^{-1}) onto local westerly jet curvature in five regions: Eastern Europe (EE), Central Asia (CA), Eastern Asia (EA), Western North America (WN), and Central North America (CN) from 1982 to 2018. Panels (**a–e**) show results based on satellite-derived NIRv estimates, and panels (**f–j**) show results from CESM1-LENS simulations. Only statistically significant trends at the 95% confidence level are displayed.

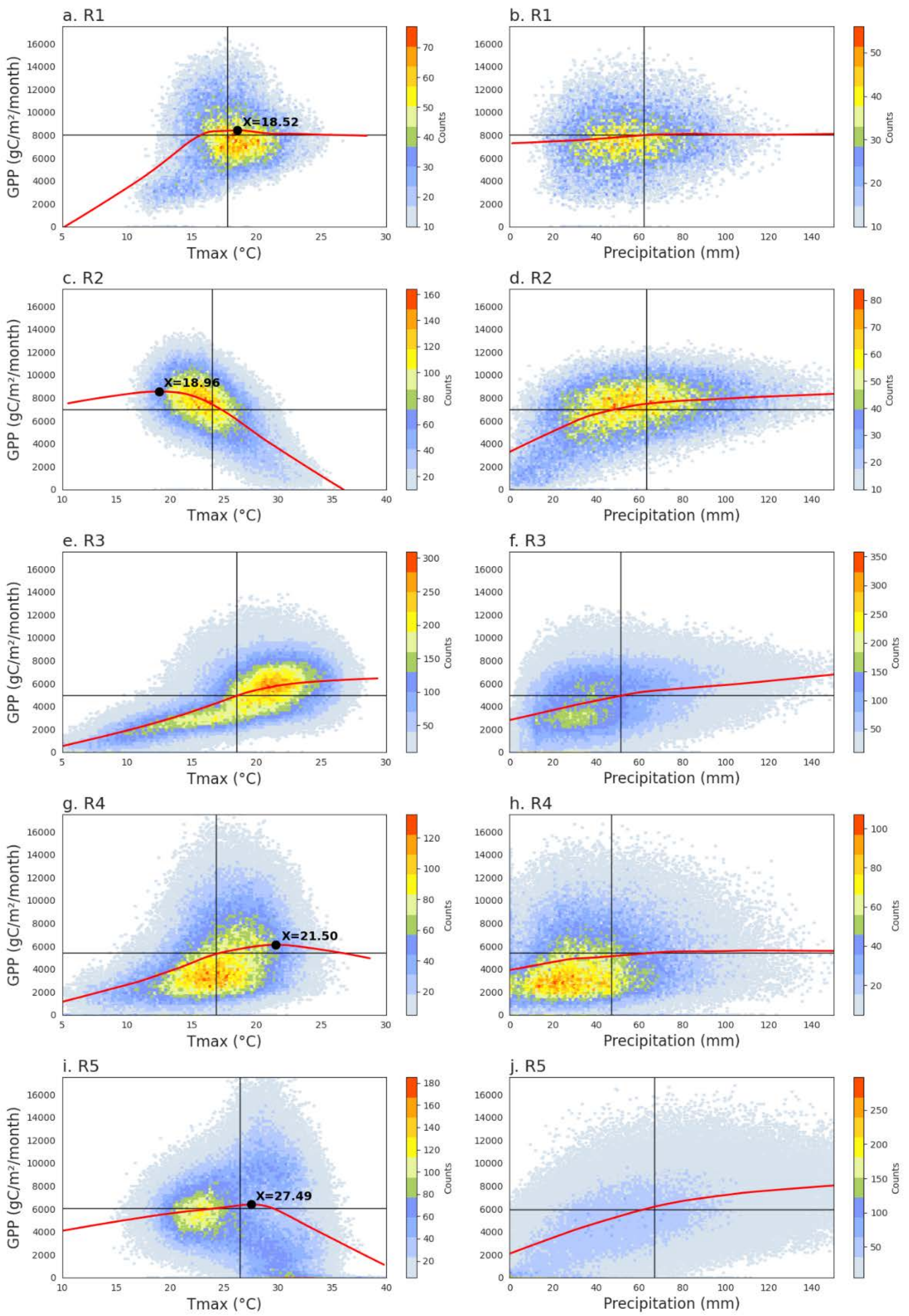

Figure S8. Response of GPP to daily maximum surface air temperature and precipitation. (**a–b**) show the joint distribution of GPP with Tmax (a) and PREC (b) in region R1. (**c–d**), (**e–f**), (**g–h**), and (**i–j**) show the same relationships for regions R2, R3, R4, and R5, respectively. In the GPP–temperature distributions, the inflection points of the GPP response curves are indicated (for only non-monotonic curves).

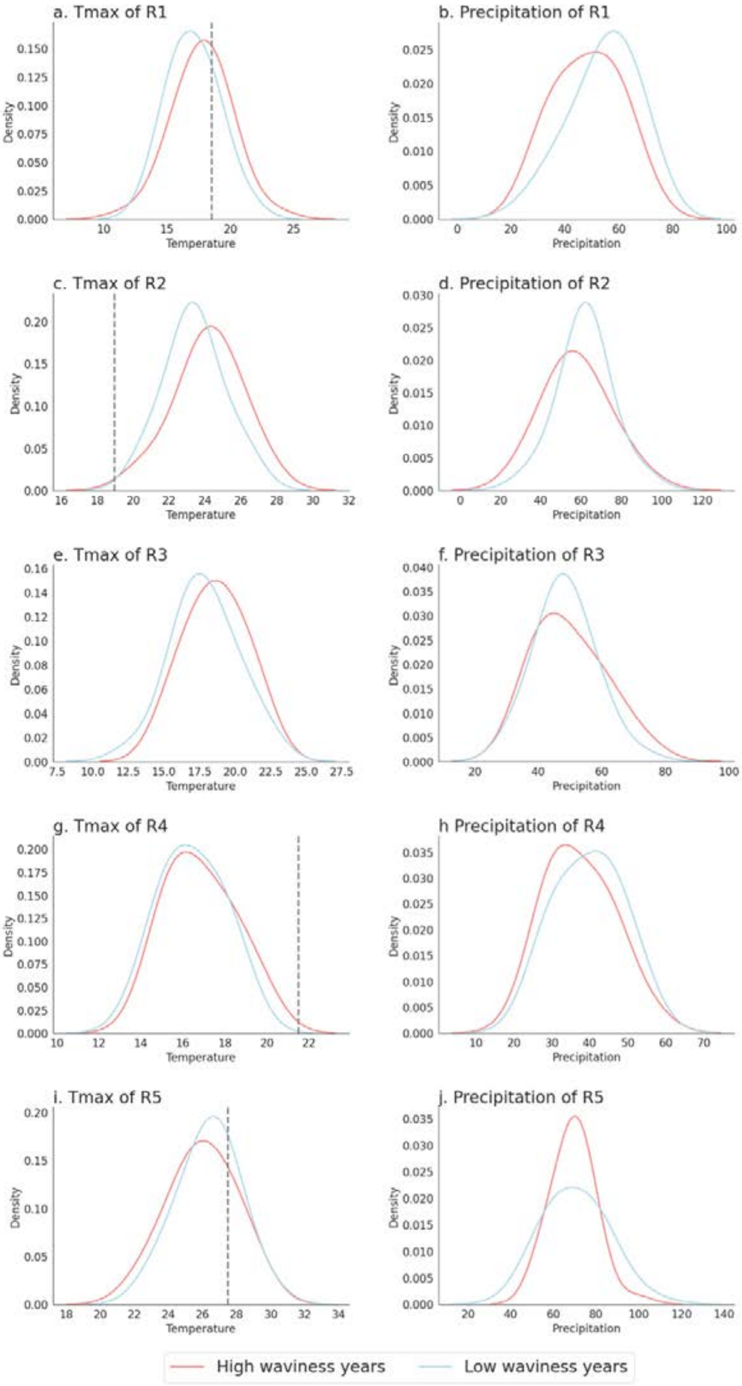

Figure S9. Relationship between meteorological elements and overall westerly curvature. Probability density distributions of daily maximum temperature (left column) and precipitation (right column) during years with high (greater than 1 standard deviation) and low (less than 1 standard deviation) overall westerly curvature. Panels (**a–b**) show R1, (**c–d**) R2, (e–f) R3, (**g–h**) R4, and (**i–j**) R5. Vertical dashed lines indicate the inflection points of the fitted GPP response curves shown in Extended Figure 7. The dashed lines in the left column indicate the inflection points of the GPP response to Tmax.

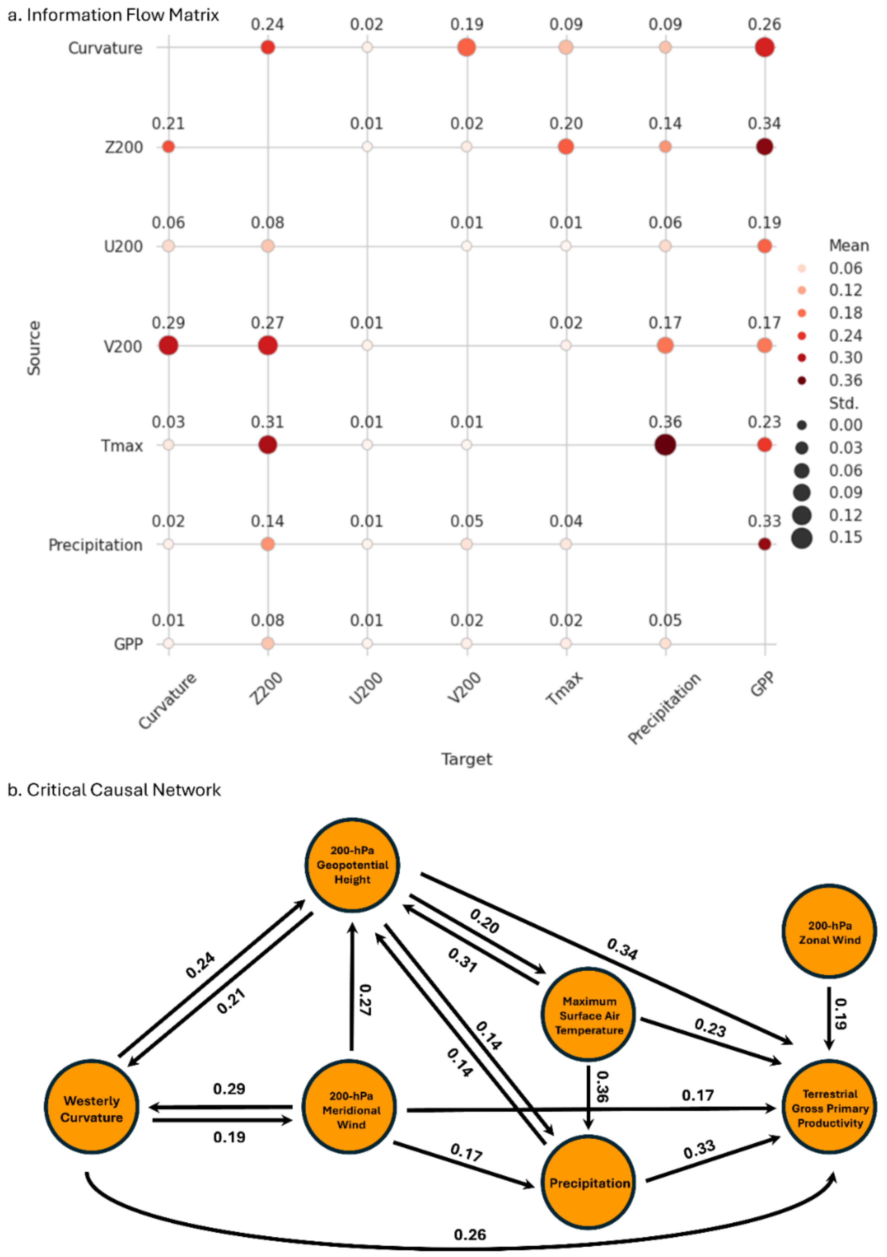

Figure S10. Causal network depicting the influence of westerly curvature on terrestrial gross primary production. (a) Standardized information flow matrix among westerly curvature, circulation factors including 200 hPa geopotential height (Z200), zonal (U200) and meridional (V200) winds, local climate variables including surface maximum temperature (Tmax) and precipitation, and gross primary production (GPP) from 1982 to 2018 (n=37). The color of each circle reflects the mean value across the five sectors (Eastern Europe, Central Asia, East Asia, Western North America, and Central North America), and the size corresponds to the standard deviation among the sectors. **(b)** The critical causal network, obtained by filtering out information flows below 0.1, represents approximately the top 60% of connections in the fully connected network.

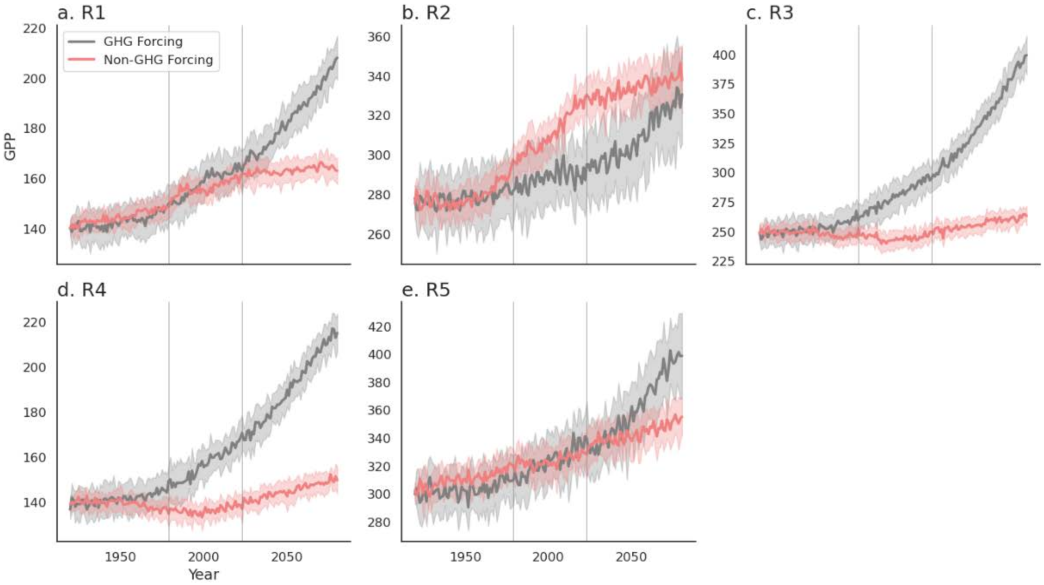

Figure S11. Projected GPP variations. Projected anthropogenic effects on Gross Primary Productivity (GPP) in regions (**a**) R1, (**b**) R2, (**c**) R3, (**d**) R4, and (e) R5. Gray lines show the impact of greenhouse gas (GHG) forcing, while red lines show the impact of non-greenhouse gas (non-GHG) forcing. Solid lines represent the multi-member ensemble mean, and shaded areas indicate the one standard deviation spread across ensemble members (n=20).

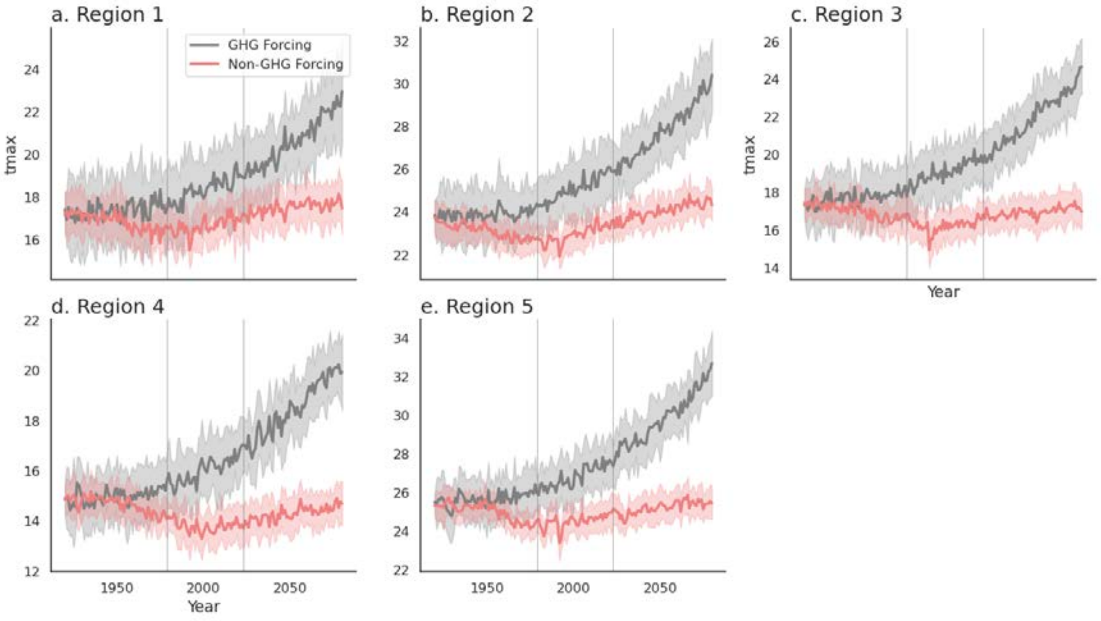

Figure S12. Projected maximum surface air temperature variations. Projected anthropogenic effects on daily maximum surface air temperature in regions (**a**) R1, (**b**) R2, (**c**) R3, (**d**) R4, and (**e**) R5. Gray lines show the impact of greenhouse gas (GHG) forcing, and red lines show the impact of non-greenhouse gas (non-GHG) forcing. Solid lines represent the multi-member ensemble mean, and shaded areas indicate the one standard deviation spread across ensemble members (n=20).

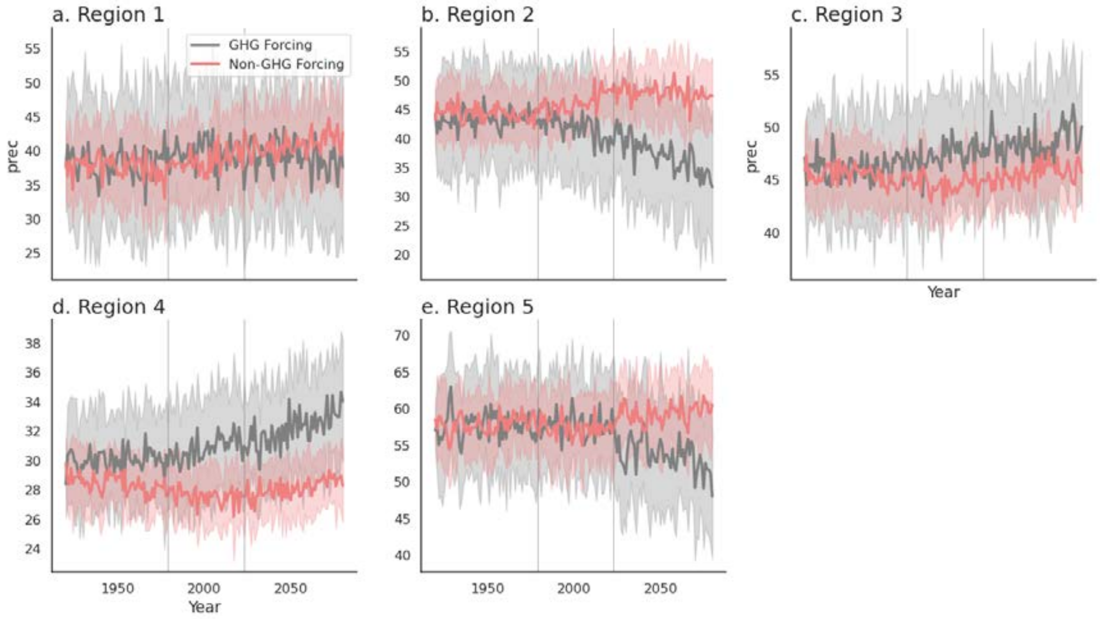

Figure S13. Projected precipitation variations. Projected anthropogenic effects on total precipitation in regions (**a**) R1, (**b**) R2, (**c**) R3, (**d**) R4, and (**e**) R5. Gray lines indicate the impact of greenhouse gas (GHG) forcing, and red lines indicate the impact of non-greenhouse gas (non-GHG) forcing. Solid lines represent the multi-member ensemble mean, and shaded areas show the one standard deviation spread across ensemble members (n=20).

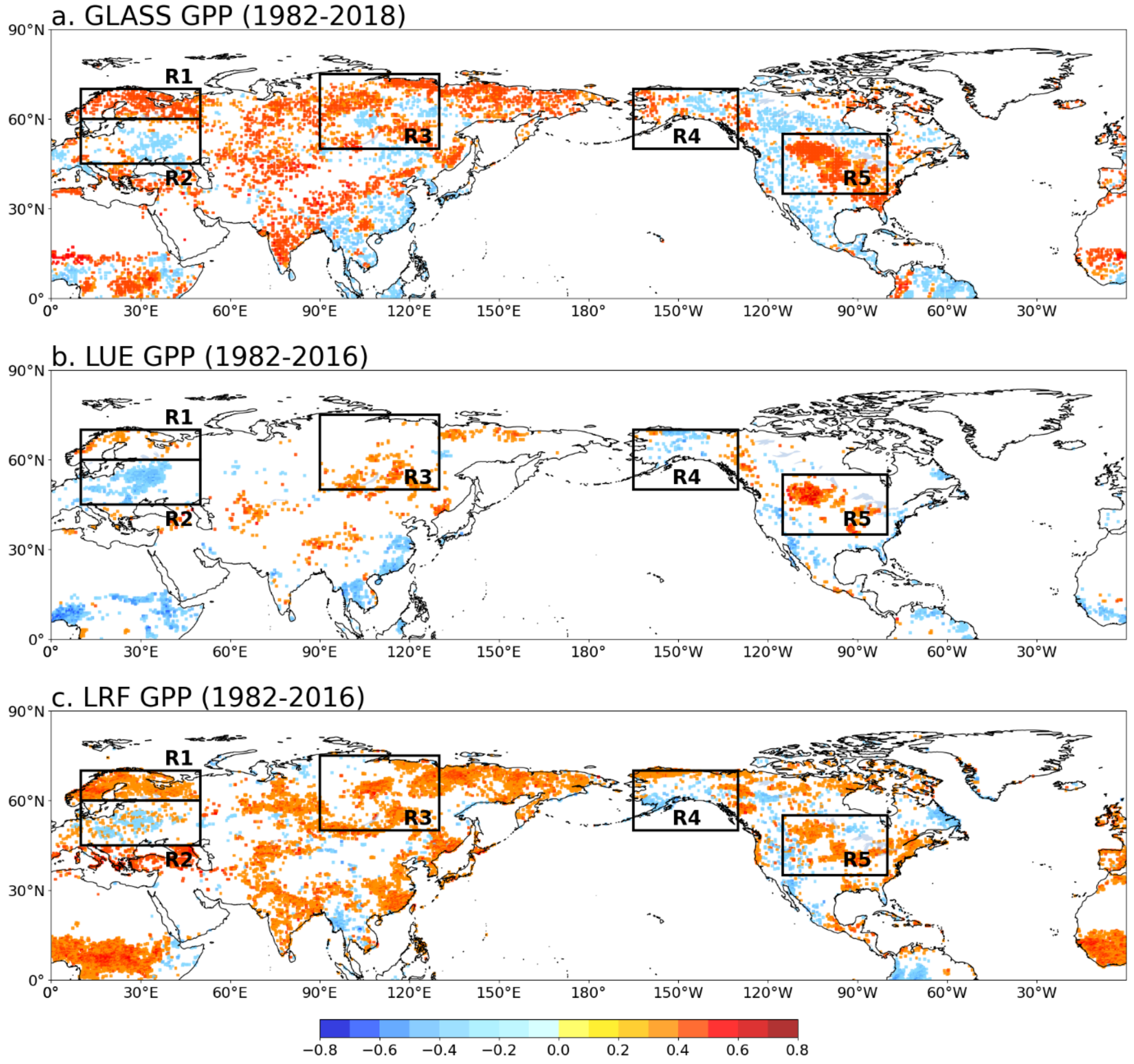

Figure S14. Relationships between summer westerly patterns in the Northern Hemisphere and gross primary productivity (GPP) based on different products. Spatial distributions of correlation coefficients between local summer GPP and Northern Hemisphere overall curvature (1982–2018) using **(a)** GLASS GPP, **(b)** LUE GPP, and **(c)** LRF GPP products. Only correlations significant at the 95% confidence level are shown.

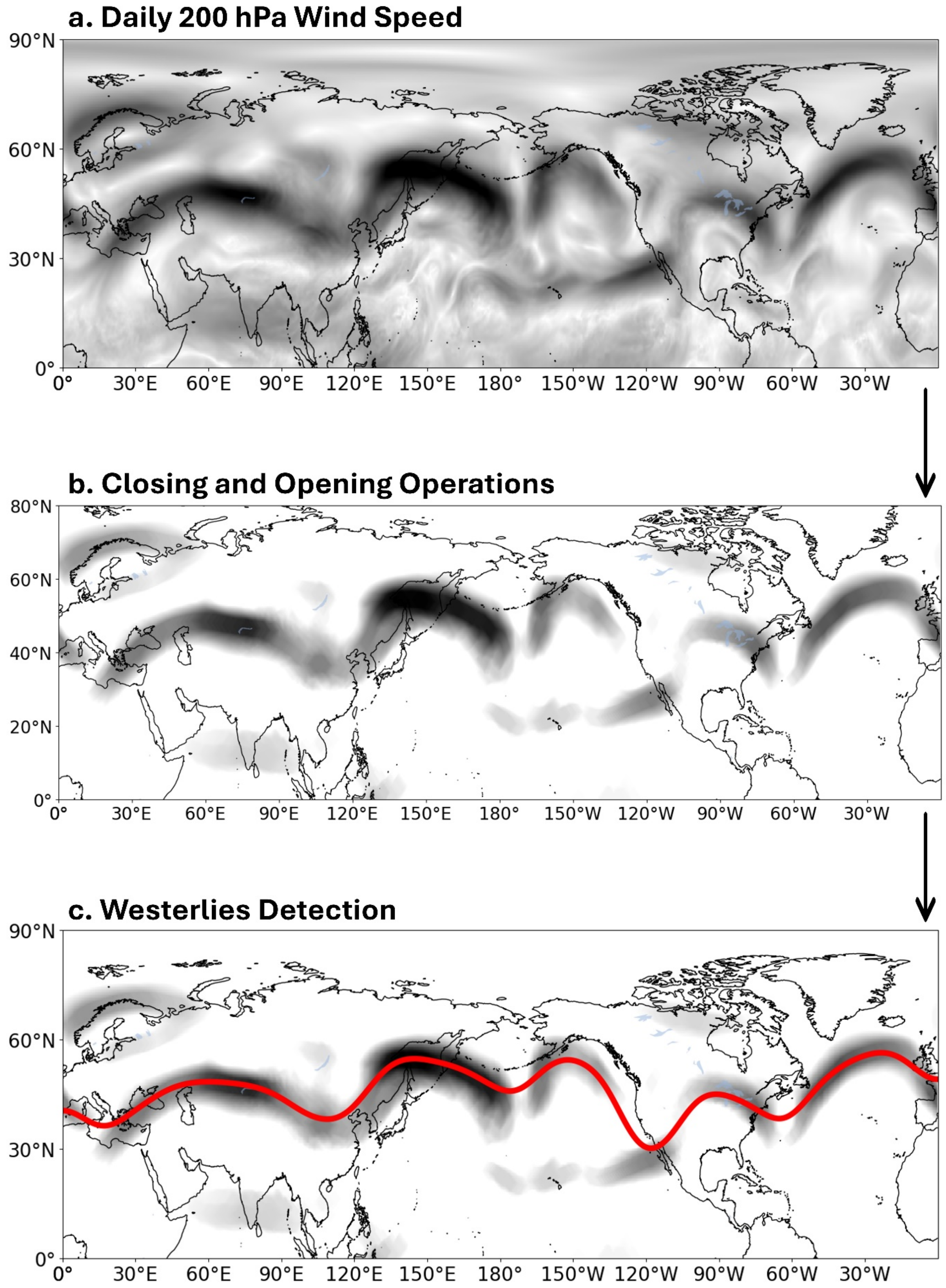

Figure S15. Schematic overview of the workflow used to detect the Northern Hemisphere westerly jet stream.

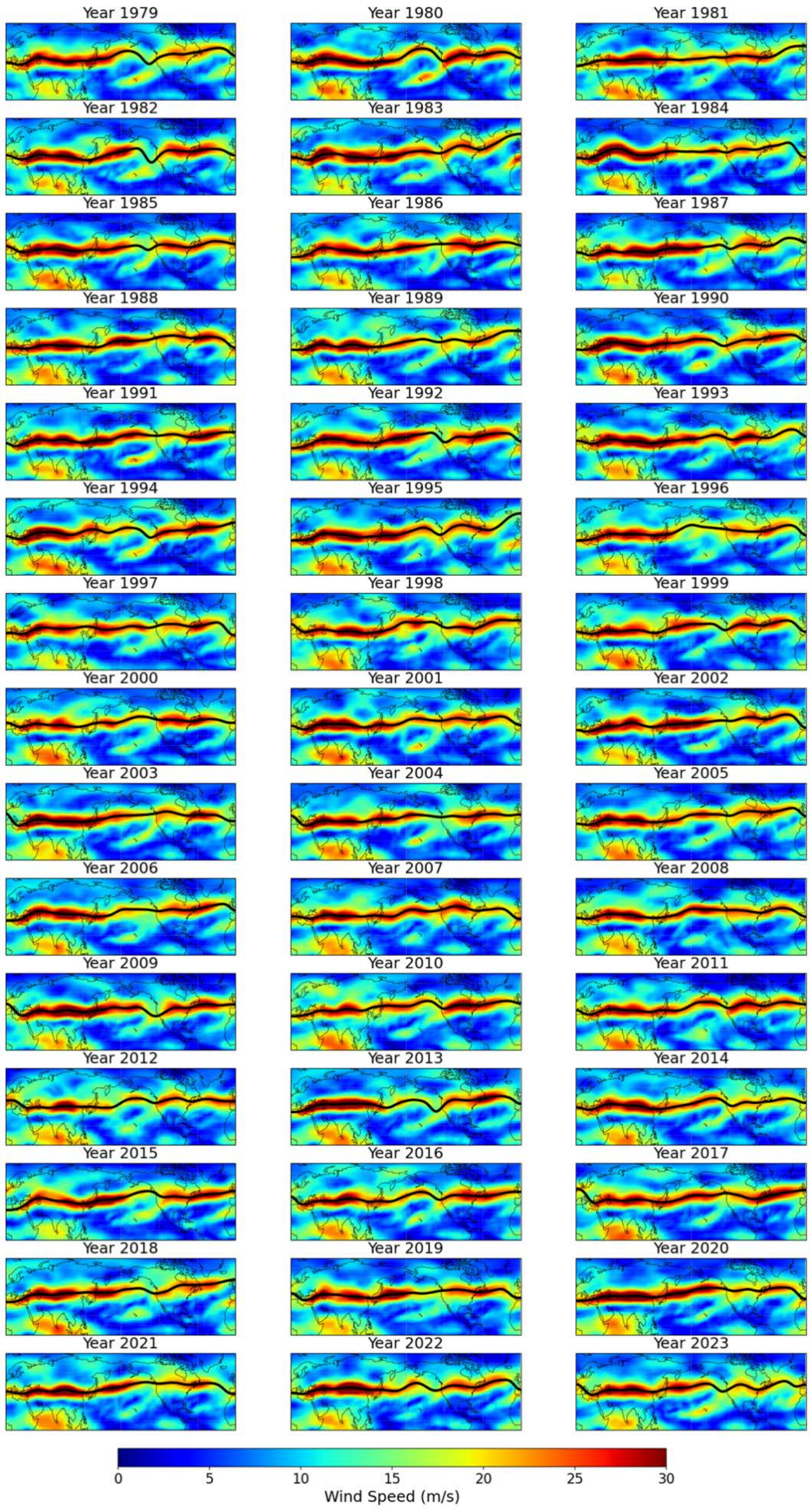

Figure S16. Annual mean 200 hPa wind speed (shading, m s^{-1}) and the corresponding detected Northern Hemisphere westerly jet axis (line) for each year from 1979 to 2023.

Supplementary References

1. Liang, X. S. Unraveling the cause-effect relation between time series. *Phys. Rev. E* **90**, 052150 (2014).
2. Rong, Y. & Liang, X. S. Panel Data Causal Inference Using a Rigorous Information Flow Analysis for Homogeneous, Independent and Identically Distributed Datasets. *IEEE Access* **9**, 47266–47274 (2021).
3. Stips, A., Macias, D., Coughlan, C., Garcia-Gorriz, E. & Liang, X. S. On the causal structure between CO2 and global temperature. *Sci Rep* **6**, 21691 (2016).
4. Sun, J. *et al*. Causal pathways underlying global soil moisture–precipitation coupling. *Nat Commun* **16**, 8935 (2025).
